# Neurophysiological Evaluation of Right-Ear Advantage During Dichotic Listening

**DOI:** 10.3389/fpsyg.2021.696263

**Published:** 2021-07-08

**Authors:** Keita Tanaka, Bernhard Ross, Shinya Kuriki, Tsuneo Harashima, Chie Obuchi, Hidehiko Okamoto

**Affiliations:** ^1^Department of Science and Engineering, Tokyo Denki University, Saitama, Japan; ^2^Baycrest Centre, Rotman Research Institute, Toronto, ON, Canada; ^3^Faculty of Health Sciences, Hokkaido University, Sapporo, Japan; ^4^Faculty of Human Sciences, University of Tsukuba, Tsukuba, Japan; ^5^Department of Speech Language and Hearing Sciences, International University of Health and Welfare, Narita, Japan; ^6^Department of Physiology, School of Medicine, International University of Health and Welfare, Narita, Japan

**Keywords:** dichotic listening, diotic listening, right-ear advantage, attention, auditory steady-state response, frequency tagging, magnetoencephalography

## Abstract

Right-ear advantage refers to the observation that when two different speech stimuli are simultaneously presented to both ears, listeners report stimuli more correctly from the right ear than the left. It is assumed to result from prominent projection along the auditory pathways to the contralateral hemisphere and the dominance of the left auditory cortex for the perception of speech elements. Our study aimed to investigate the role of attention in the right-ear advantage. We recorded magnetoencephalography data while participants listened to pairs of Japanese two-syllable words (namely, “/ta/ /ko/” or “/i/ /ka/”). The amplitudes of the stimuli were modulated at 35 Hz in one ear and 45 Hz in the other. Such frequency-tagging allowed the selective quantification of left and right auditory cortex responses to left and right ear stimuli. Behavioral tests confirmed the right-ear advantage, with higher accuracy for stimuli presented to the right ear than to the left. The amplitude of the auditory steady-state response was larger when attending to the stimuli compared to passive listening. We detected a correlation between the attention-related increase in the amplitude of the auditory steady-state response and the laterality index of behavioral accuracy. The right-ear advantage in the free-response dichotic listening was also found in neural activities in the left auditory cortex, suggesting that it was related to the allocation of attention to both ears.

## Introduction

The asymmetry of hemispheric organization of brain function is a major topic of research. Hemispheric asymmetry in audition is even more complicated. It is known that the left hemisphere plays a role in language function. Additionally, projections from each ear to the bilateral auditory cortices are commonly asymmetric (Hakvoort et al., 2016; Mei et al., 2020). The contralateral pathway is stronger than the ipsilateral pathway (Rosenzweig, 1951; Hiscock and Kinsbourne, 2011). In particular, during dichotic listening (DL) to speech stimuli, listeners reported stimuli more correctly from the right than the left ear. This preference for the right ear is termed right-ear advantage (REA) during DL. The structural model originally suggested by Kimura (1961a,b) is the most widely accepted explanation for REA. This model associates the REA with the combined effect of specialization of the left hemisphere for language processing and contralateral dominance of the auditory pathway (Kimura, 1967).

Listeners use selective attention to focus on auditory information from the right and left ears without moving their heads. The structural model of the REA does not incorporate the fact that attention may play a role in right ear bias, as shown in a recent study (Payne et al., 2017). Additionally, neurophysiological results using directed DL in REA suggest the involvement of selective attention (Jäncke et al., 2001; Alho et al., 2012; Payne et al., 2017). The sustained field of the event-related potential was stronger in the left auditory cortex (AC) than in the right in both non-instruction and right-ear attention conditions (Alho et al., 2012). Payne et al. (2017) also revealed that significant increase of alpha power in the parietal and right frontal-parietal areas during right-ear attention conditions. Jäncke et al. (2001) used functional magnetic resonance imaging (fMRI) and demonstrated that activity in the left AC increased when selectively attending to right-ear sounds, and vice versa, right hemispheric activity increased when attending to the left-ear sounds. These studies used neurophysiological responses as an index of selective attention. In previous studies on the REA, directed DL tasks were used in which participants were instructed to pay attention to the left or right ear. Alternatively, Hugdahl and Andersson (1986) detected the REA in a free-report (non-forced-attention) condition. In the non-forced-attention condition, participants are required to allocate attention to both ears and need attention, which is considered endogenous (top-down). Therefore, the REA is indicated if the accuracy for the right-ear stimuli is higher than that for the left-ear stimuli.

The current study aims to examine both the structural model and the attentional bias model in REA using neurophysiological methods with magnetoencephalography (MEG). In particular, for the attention bias model, we quantitatively evaluated the allocation of attention of participants. In structural models, separate observations of left and right-ear responses to different acoustic stimuli, such as tones and speech sounds, are useful for studying hemispheric differences in auditory function (Tononi et al., 1998; Zatorre and Belin, 2001; Fujiki et al., 2002; Bharadwaj et al., 2014). Although contralateral projections are dominant in the auditory pathway, ipsilateral projections also exist. The responses obtained from the left and right auditory cortices consist of a mixed response to ipsilateral and contralateral inputs. It is difficult to separate the AC activities elicited by concurrent sound inputs to the left and right ears. Thus, we used a frequency-tagging method and MEG to investigate the functions of attention bias and brain asymmetry. Electroencephalogram recordings of evoked responses from the left and right AC appear superimposed at frontocentral electrodes, making it difficult to separate the signal sources. However, MEG has a high spatial resolution. In addition, the magnetic field is rotated by 90 degrees in relation to the electric field, thereby helping to distinguish left and right AC activities. We labeled the inputs from the left and right ears by tagging the stimulus sounds with amplitude modulations of different frequencies at each ear and measuring auditory steady-state responses (ASSR) of cortical origin for each modulation frequency.

We attempted to quantitatively evaluate the allocation of attention during the DL using ASSR, which reflects the activity of the AC. ASSRs to sinusoidal amplitude-modulated tones and repeated click sounds have been used extensively. Commonly, response amplitudes are maximal when the modulation frequency and click repetition rates are ~40 Hz (Galambos et al., 1981; Mäkelä and Hari, 1987; Hari et al., 1989; Ross et al., 2000; Tanaka et al., 2013). Notably, 40-Hz ASSRs are closely related to gamma oscillations in thalamocortical networks (Plourde et al., 1998; Jones, 2002). Several studies have documented this network (Destexhe et al., 1998; Plourde et al., 1998; Jones, 2002; Llinas et al., 2002) and Ross and Fujioka (2016) depicted the networks as a diagram [Figure 11 in Ross and Fujioka (2016)]. The gamma oscillation networks consist of two circuits, one representing sensory input, called the specific sensory circuit, and a second loop associated with higher-order representation referred to as the non-specific binding circuit. The 40-Hz ASSR is formed primarily by specific circuit activity, which is associated with bottom-up processing and modulates ASSRs based on variations in stimulation properties. Conversely, an attention function may be more closely related to a non-specific binding circuit. Therefore, by observing the activity of specific sensory and the non-specific binding circuits in the gamma oscillation at 40 Hz, it may be possible to determine which circuit contributes to the REA in DL. In this study, we examined the effect of non-specific binding circuit activity as “ASSR modulation.” Notably, the rightward attention in DL would display the behavior of the non-specific binding circuit.

Previous studies on REA have typically used a DL task in which participants were instructed to direct their attention to the left or right ear (Jäncke et al., 2001; Alho et al., 2012; Payne et al., 2017). However, due to its ease and high accuracy, this method demonstrated a ceiling effect and could not accurately evaluate brain activity due to allocation of attention. Therefore, in this study, we performed a free-response DL task (non-instruction) using meaningful Japanese two-syllable words. This DL task was more difficult than the directed DL task, thus permitting clear and quantitative visualization of the correlation between the accuracy of each left or right ear and activity in the left or right auditory cortex. In addition, we also performed a diotic listening task (identical for the two ears), where participants were presented with the same speech stimulus to both ears. Even when the same speech stimulus is presented to the left and right ears in diotic listening, it is speculated that the attentional bias may be activated in the same way as in the DL task in order for participants to answer.

We hypothesized that if the structural model in the REA was involved, the ASSR amplitude would reflect the combined effect of left hemisphere dominance for language processing and contralateral dominance of the auditory pathway, while if the attentional bias model was involved, the ASSR modulation would reflect the effect of allocation of attention. Furthermore, since the same speech stimulus is presented to both ears in the diotic listening, it is not possible to examine from their behavior which ear the participants referred to in their responses. However, if a correlation is observed between the accuracy in the diotic listening task and the ASSR modulation, it should be possible to estimate right ear or left ear use from the brain activity. Therefore, our results will add to the understanding of the REA in DL.

## Materials and Methods

### Participants

Eighteen Japanese male adults (mean 22.9 years, standard deviation (SD) = 4.7) participated in this study. Participants were all undergraduate or graduate students at Japanese universities who were native speakers of Japanese. All participants were right-handed and had no history of otolaryngological or neurological disorders. All participants provided written consent after being informed of the nature of the study. All procedures were performed in accordance with the Declaration of Helsinki and were approved by the Research Ethics Committee of Tokyo Denki University.

### Stimuli and Experimental Design

We prepared two lists of 48 meaningful Japanese two-syllable words spoken in a female voice and created word pairs (namely, “/ta/ /ko/” means “octopus” or “/i/ /ka/” means “squid”) by sampling single items from both lists. The stimulus words were spoken for a duration of 330–503 ms. When applicable, a silent interval was added to create sound samples of a consistent length of 500 ms. The speech sounds were amplitude-modulated at 35 Hz and 45 Hz, with a modulation depth of 100%. We simultaneously presented stimulus sounds to the left and right ears at an inter-stimulus interval of 3 s at an intensity of 90 dB SPL.

Stimulus timing was controlled using Presentation software (Neurobehavioral Systems, Berkeley, CA, USA). Stimulus sounds were presented with insert earphones (E-A-RTONE 3A, Aearo Company Auditory Systems, Indianapolis, IN, USA) through a 1.5-m plastic tube attached to foam earpieces (E-A-RLINK, Aearo Company Auditory Systems, Indianapolis, IN, USA).

The experiments consisted of active and passive conditions. In the active condition, participants were instructed to write down the two-syllable sounds they heard in the left and right ears during the 3 s inter-stimulus interval on a prepared response sheet. In the passive condition, participants watched a silent movie during stimulation as a control condition. The same participants performed both active and passive conditions.

In addition, a diotic listening task was performed for comparison with DL. We counter-balanced word lists and modulation frequencies to eliminate word dependencies and the effects of modulation frequency during both DL and diotic listening tasks.

**Figure 1:**
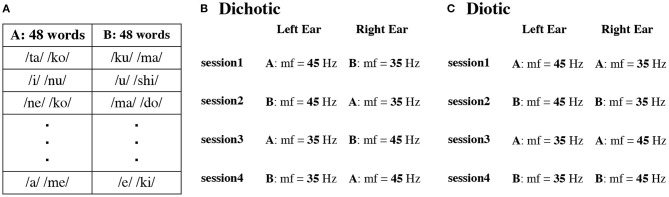
Example of two sets (Set A and B) with different presentation order of 48 words in each participant **(A)**. Presentation procedure in the dichotic **(B)** and diotic **(C)** listening tasks. mf indicates modulation frequency.

The DL task consisted of four sessions (Figure 1B). Stimulus sequences were individually prepared for each participant. First, we prepared 48 word pairs by permuting the word sets (A group words and B group words, Figure 1A). In the first session, we presented words modulated at 45 Hz to the left ear and words modulated at 35 Hz to the right ear. In the second session, we randomized the word pairs, and the modulation frequency was 45 and 35 Hz at the same frequency as in session one. In the third and fourth sessions, modulation frequencies were switched. The word pairs were randomly chosen in each session and each participant in the DL task. The same pair of the first syllable on the left and right ears was 2.44% on average for each participant.

In contrast, in the diotic listening test (Figure 1C), we presented the same speech stimuli to the left and right ears. The word sequence was randomly changed in each session and for each participant. The same schema of amplitude modulations, as for the DL condition, was used. In session one and three, words from list A were presented and in sessions two and four from list B.

Participants first performed the active condition of the DL task, followed by the passive condition, followed by a 10-min break. They then performed the active condition of the diotic listening task, followed by the passive condition on the same day.

### MEG Recording

MEG acquisition was performed in a magnetically shielded and acoustically quiet room using a 204-channel whole-head planar-type gradiometer MEG system (Vector-view, ELEKTA, Neuromag, Helsinki, Finland) at the National Institute for Physiological Sciences (NIPS) in Okazaki. Before the MEG recordings, five head position indicator coils were attached to the participant's scalp, and a 3D digitizer (Polhemus Inc., Colchester, VT) was used to record the positions of the coils, three anatomical landmarks, including the nasion, bilateral pre-auricular points, and the head shape. A current was passed through the five head position indicator coils, and the resulting magnetic fields were used to evaluate the head position with reference to the MEG sensor. MEGs were filtered using a bandpass of 0.1–200 Hz and digitized at 1 kHz. Participants were comfortably seated upright during MEG measurements.

### Data Analyses

MEG data analysis was performed using the *Brainstorm* Matlab toolbox (http://neuroimage.usc.edu/brainstorm). Epochs were defined as the period from −500 ms to 1000 ms relative to the stimulus onset time (*t* = 0). Epochs with amplitude values >3 pT/cm were excluded from the averaging as artifact-contaminated epochs. First, we combined the averaged wave forms from the first and second sessions for each sensor. An estimate of noise covariance was computed based on the baseline interval between −500 ms and zero. To detect 35-Hz and 45-Hz ASSRs, we applied bandpass filters at 32–37 Hz and 42–47 Hz to the averaged waveforms in the time interval of −500 ms and 1000 ms. To obtain the cortical current distribution of 35-Hz and 45-Hz ASSRs (Appendix 1), we used minimum norm estimates (Hämäläinen and Ilmoniemi, 1994) using the *Brainstorm* default parameters. Head modeling within *Brainstorm* used 15,000 elementary current sources constrained to the cortical mantle to sample the brain surface. The magnetic source activities were calculated using a single-sphere head model and constraining the orientations of the sources to be normal to the cortical surface. After estimating the current density map of the brain model from the 35-Hz and 45-Hz ASSR, regions of interest were selected in Heschl's gyrus of the primary AC using the AAL atlas (Montreal Neurological Institute: http://www.gin.cnrs.fr/en/tools/aal-aal2/). Heschl's gyrus is the source of the ASSR, and Penna et al. used neuromagnetic activity in the Heschl's gyrus to explain the “structural model” of the REA in DL task (Penna et al., 2007).

Time courses of the amplitude of the ASSR at 35 Hz and 45 Hz were obtained as the absolute value and angle of a Hilbert transform that was applied to the source waveforms to the left and right ears in the left and right AC. Finally, we reduced the effect of frequency dependency from the ASSR by averaging the mean Hilbert amplitude of 35-Hz ASSR (from sessions 1 and 2) and 45-Hz ASSR (from sessions 3 and 4) between 1 and 500 ms. The result defined the ASSR amplitude for the left ear stimuli in the left or right AC. Similarly, the mean Hilbert amplitude of the 45-Hz ASSR (from sessions 1 and 2) and the mean Hilbert amplitude of 35-Hz ASSR (from sessions 3 and 4) defined the ASSR amplitude for the right-ear stimuli in the left or right AC. The ASSR amplitude was evaluated by a three-way repeated-measures analysis of variance (ANOVA) using conditions (active vs. passive), ears (left vs. right ears), and hemispheres (left vs. right AC) as factors. Thereafter, repeated measures Tukey multiple comparison tests were performed for multiple comparisons. Statistical results were considered significant at *p* = 0.05. All analyses were performed using the customized MATLAB software.

ASSRs during the passive condition mainly involve a specific circuit in gamma oscillation networks (Ross and Fujioka, 2016). Participants watched a silent movie during stimulation. Alternatively, in the active condition, participants were required to pay attention to the sounds, and thus, ASSRs in the active condition involve specific and non-specific binding circuits. Therefore, to detect the amplitude reflecting the increment of the behavior of the non-specific binding circuit, we calculated ASSR modulation using the following formula: ASSR modulation = active condition/passive condition. We applied the formula to the ASSR amplitude in DL and diotic listening tasks. Here, an ASSR modulation value >1 indicates an increase in ASSR amplitude in the active condition as compared to the passive condition. The ASSR modulation was evaluated by a two-way repeated-measures ANOVA using ears (left vs. right ears) and hemispheres (left vs. right AC) as factors. Thereafter, repeated measures Tukey multiple comparison tests were performed for multiple comparisons.

We calculated individual laterality indices (LI) for accuracy as the difference between the right and left ears, the LI of accuracy = (right ear – left ear)/(right ear + left ear). Thus, the LI was +1 for accuracy completely lateralized to the REA, zero for symmetrical accuracy, and −1 for accuracy completely lateralized to the left ear advantage. A subsequent analysis of covariance (ANCOVA) was performed to determine if the left and right ACs differed.

## Results

### Behavioral Results

**Figure 2:**
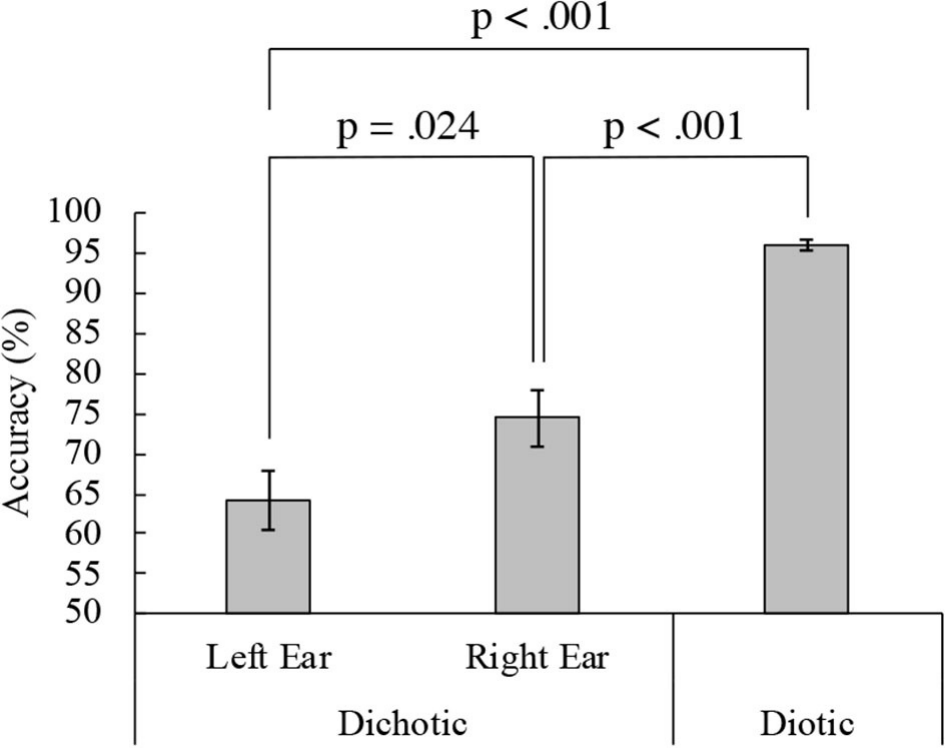
Dichotic and diotic task accuracy. Mean accuracies from 18 participants in the left (64.15%) and right (73.61%) ears in the dichotic and diotic (96.44%) listening tasks. Error bars indicate standard error of the mean.

Figure 2 shows the mean accuracy in the DL and diotic listening tasks for the active condition. The mean accuracy was 64.15% (standard deviation, SD = 15.72) for the left ear and 73.61% (SD = 14.93) for the right in the DL task. Therefore, REA was observed in the behavioral performance. Conversely, the mean accuracy during the diotic listening task was 96.44% (SD = 2.96). Accuracy significantly differed between the left and right ears in the DL and the diotic listening tasks {one-way ANOVA, [*F*_(2, 34)_ = 47.29, *p* < 0.001]}. Ryan's method for multiple comparisons indicated that the diotic listening task accuracy was significantly higher than the DL task accuracy (p < 0.001). Additionally, in the DL task, the accuracy of the right ear was significantly higher than that of the left ear (*p* = 0.024). The results of a paired *t*-test also revealed that the difference was significant [t(17) = 2.54, *p* = 0.021]. A two-way (modulation frequencies, ears) ANOVA was performed. There was no significant main effect between the 35-Hz and 45-Hz of the modulation frequency. {Left ear 35-Hz: 67.82% (SD = 16.74), 45-Hz: 63.95% (SD = 17.12), Right ear 35-Hz: 71.06% (SD = 15.03), 45-Hz: 76.16% (SD = 17.62), main effect: modulation frequencies: [*F*_(1, 17)_ = 0.35, *p* = 0.563], ears: [*F*_(1, 17)_ = 6.45, *p* = 0.021], interaction effect: modulation frequencies and ears: [*F*_(1, 17)_ = 3.81, *p* = 0.068]}.

### ASSR Amplitude

**Figure 3:**
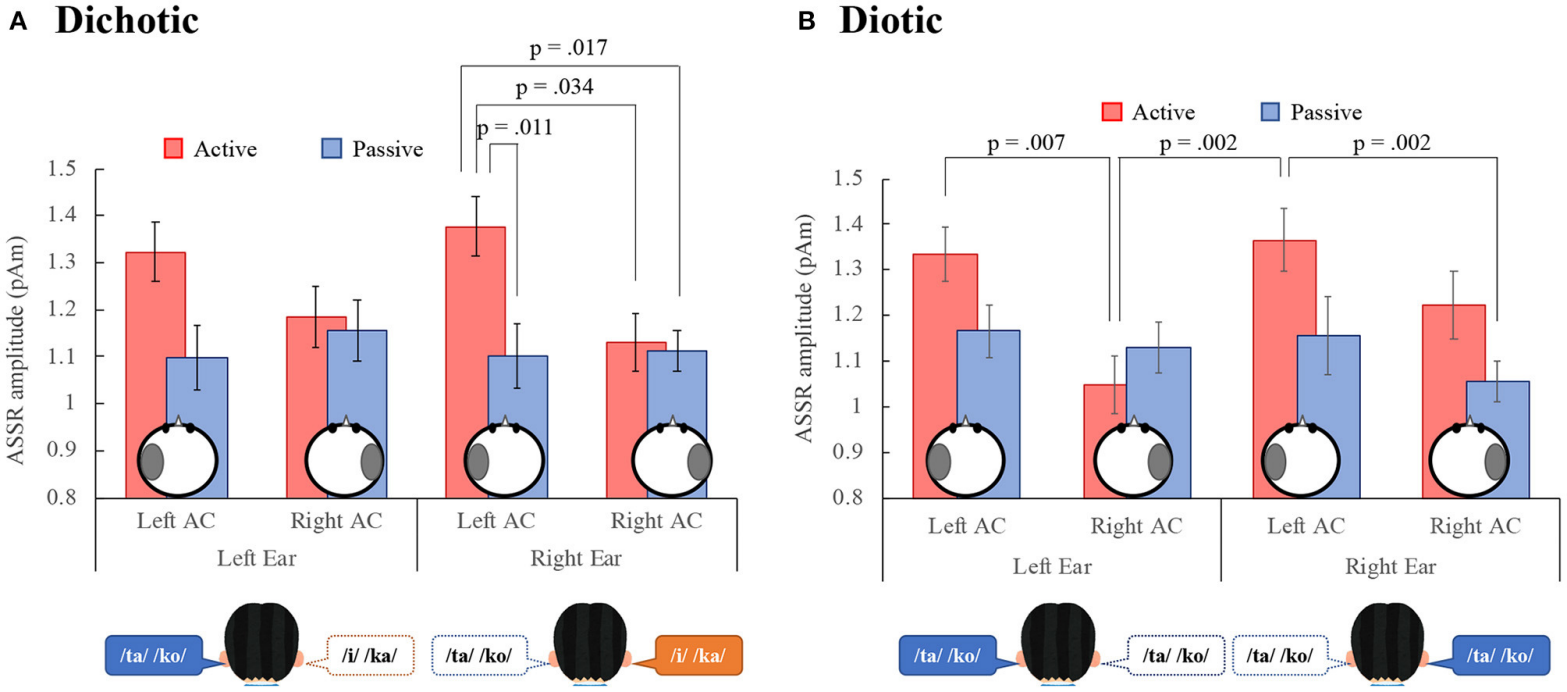
Grand-mean values across 18 participants of the ASSR amplitude in the left and right auditory cortex (Left AC or Right AC) to the left and right-ears during the dichotic **(A)** and diotic **(B)** listening tasks. Error bars indicate standard error of the mean. ASSR, auditory steady-state response; AC, auditory cortex.

Figure 3 shows the grand mean ASSR amplitudes between 0 and 500 ms for all participants in the left and right AC to the left and right ears in the DL and the diotic listening tasks. For the DL task (Figure 3A), a repeated measures ANOVA with the within-participant factor conditions (active vs. passive), ears (left vs. right ears), and hemispheres (left vs. right AC), revealed a significant main effect of conditions, indicating that the ASSR amplitude in the active condition was significantly larger than in the passive condition. There was also a significant interaction between the conditions and hemispheres {main effect: conditions: [*F*_(1, 17)_ = 5.34, *p* = 0.033], ear: [*F*_(1, 17)_ = 0.09, *p* = 0.762], hemispheres: [*F*_(1, 17)_ = 3.90, *p* = 0.065], interaction effect: conditions and hemispheres: [*F*_(1, 17)_ = 12.51, *p* = 0.003], conditions and ears: [*F*_(1, 17)_ = 0.09, *p* = 0.767], conditions, hemispheres, and ears: [*F*_(1, 17)_ = 0.36, *p* = 0.555]}. A repeated measures Tukey multiple comparison test revealed for right ear stimuli that the ASSR amplitude in the left AC was significantly larger during the active than the passive condition (*p* = 0.011), and was larger than the right AC amplitude during the active (*p* = 0.034) and passive (*p* = 0.017) conditions. These results indicate that the ASSR amplitude was modulated by the DL task and may reflect left hemispheric dominance in the REA.

In the diotic listening task (Figure 3B), the ANOVA revealed a significant main effect of conditions, indicating that the amplitude in the active condition was significantly larger than in the passive condition. Furthermore, the main effect of hemisphere was significant, indicating that the amplitude in the left AC was larger than that in the right AC. There was a significant interaction effect between the conditions and ears, and the conditions and hemispheres {main effect: conditions: [*F*_(1, 17)_ = 7.38, *p* = 0.015], ears: [*F*_(1, 17)_ = 0.57, *p* = 0.460], hemispheres: [*F*_(1, 17)_ = 11.13, *p* = 0.004], interaction effect: conditions and hemispheres: [*F*_(1, 17)_ = 5.22, *p* = 0.036], conditions and ears: [*F*_(1, 17)_ = 4.79, *p* = 0.043], hemispheres and ears: [*F*_(1, 17)_ = 0.23, *p* = 0.640], conditions, hemispheres, and ears: [*F*_(1, 17)_ = 2.18, *p* = 0.159]}. A repeated measures Tukey multiple comparison test revealed that in the active condition, the ASSR amplitude in the left AC was significantly larger than that in the right AC to the left ear (*p* = 0.007) and the amplitude in the left AC to the right ear was significantly larger than that in the right AC to the left ear (*p* = 0.002). Additionally, in the right ear, the amplitude in the left AC during the active condition was significantly larger than that in the right AC during the passive condition (*p* = 0.002).

We also performed a four-way [dichotic vs. diotic (tasks), conditions, ears, hemispheres] ANOVA to compare ASSR amplitudes during the DL and the diotic listening tasks. The results showed that there was no significant main effect between the DL and the diotic listening tasks {main effect: tasks: [*F*_(1, 17)_ = 0.00, *p* = 0.987], conditions: [*F*_(1, 17)_ = 8.39, *p* = 0.01], ear: [*F*_(1, 17)_ = 0.28, *p* = 0.601], hemispheres: [*F*_(1, 17)_ = 9.48, *p* = 0.007], interaction effect: tasks and conditions: [*F*_(1, 17)_ = 0.15, *p* = 0.708], tasks and ears: [*F*_(1, 17)_ = 0.41, *p* = 0.529], tasks and hemispheres: [*F*_(1, 17)_ = 2.26, *p* = 0.151], conditions and ears: [*F*_(1, 17)_ = 2.43, *p* = 0.138], conditions and hemispheres: [*F*_(1, 17)_ = 12.51, *p* = 0.003], ears and hemispheres: [*F*_(1, 17)_ = 0.12, *p* = 0.729], tasks, conditions, and hemispheres: [*F*_(1, 17)_ = 2.74, *p* = 0.116], tasks, conditions, and hemispheres: [*F*_(1, 17)_ = 1.25, *p* = 0.280], conditions, ears, and hemispheres: [*F*_(1, 17)_ = 0.86, *p* = 0.366], tasks, conditions, ears, and hemispheres: [*F*_(1, 17)_ = 2.02, *p* = 0.174]}.

### ASSR Modulation

**Figure 4:**
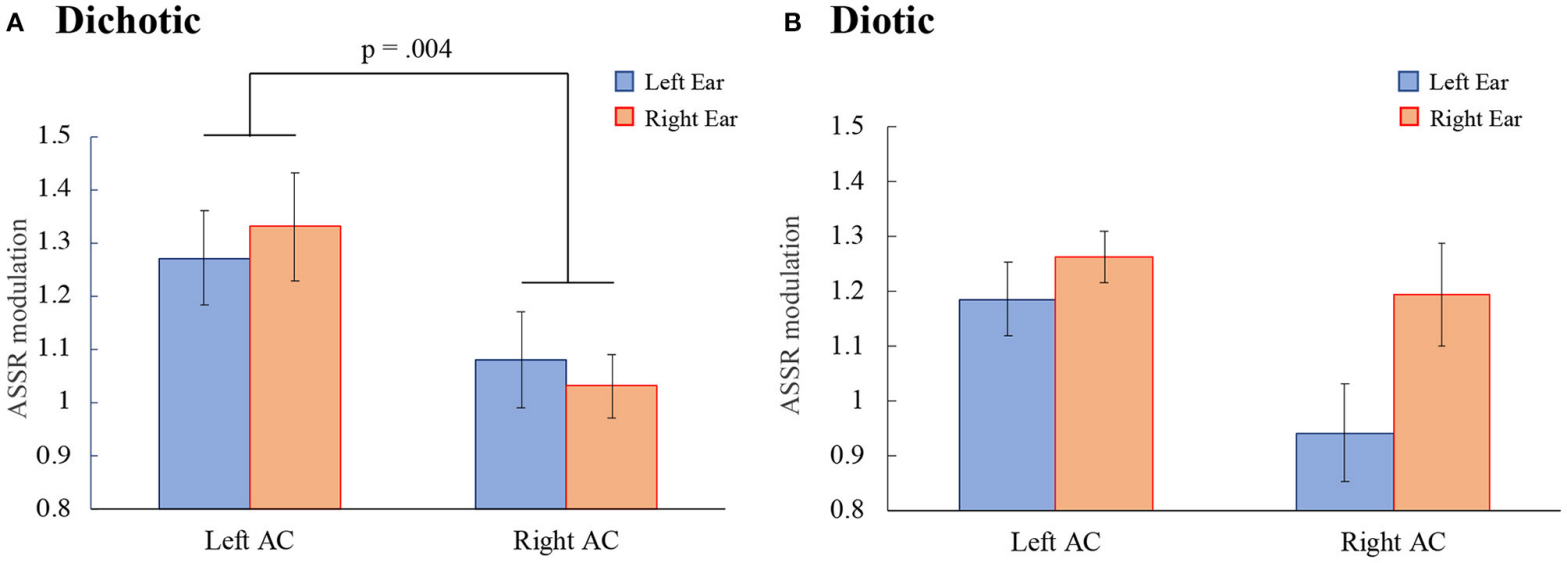
The ASSR modulations in the left and right auditory cortex (Left AC or Right AC) to the left and right ears during the dichotic **(A)** and the diotic **(B)** listening tasks. Error bars indicate standard error of the mean. ASSR, auditory steady-state response; AC, auditory cortex.

Figure 4 shows the ASSR modulation in the DL and diotic listening tasks. In the DL task (Figure 4A), a repeated measures ANOVA with the within-subject factors ears (left- vs. right ears) and hemispheres (left vs. right AC) revealed a significant main effect of hemispheres, indicating that the ASSR modulation in the left AC was larger than in the right AC. No significant main effect of ears or interactions were observed {main effects: ears: [*F*_(1, 17)_ = 0.00, *p* = 0.955], hemispheres: [*F*_(1, 17)_ = 11.10, *p* = 0.004], interaction effect: [*F*_(1, 17)_ = 1.11, *p* = 0.306]}.

In the diotic listening task (Figure 4B) no significant main effects or interactions were observed {main effects: ears: [*F*_(1, 17)_ = 3.56, *p* = 0.076], hemispheres: [*F*_(1, 17)_ = 2.43, *p* = 0.138]; interaction effect: [*F*_(1, 17)_ = 2.40, *p* = 0.139]}.

**Figure 5:**
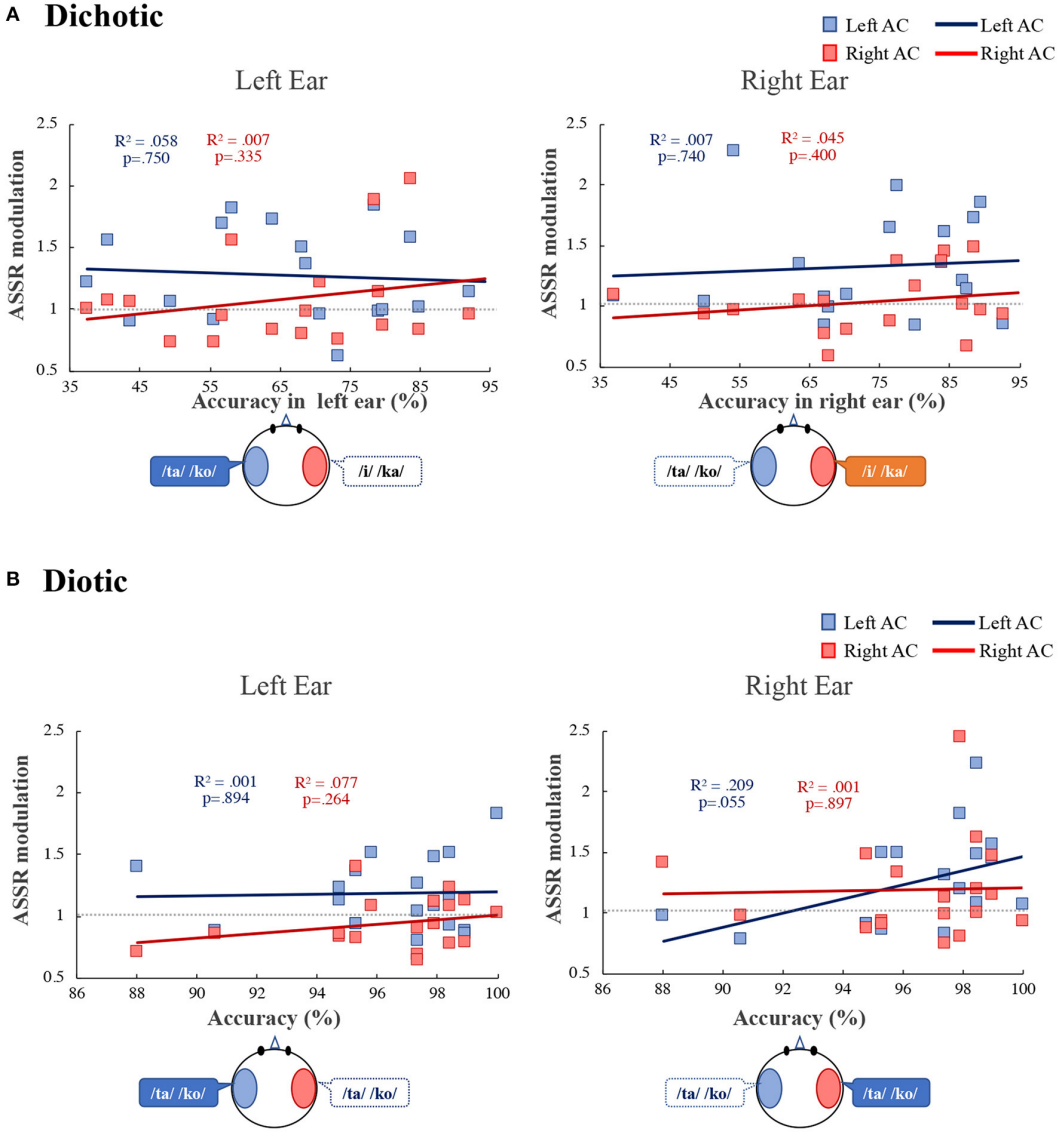
Correlation between the accuracy and the ASSR modulation in the left (blue) and right (red) auditory cortex (Left AC or Right AC) to the left and right-ears during the dichotic listening **(A)** diotic listening **(B)** tasks. *R*^2^ indicates determination coefficient. ASSR, auditory steady-state response; AC, auditory cortex.

Additionally, we investigated the relationship between participant behavior and ASSR modulation. We found a correlation between ASSR modulation and accuracy during the diotic listening and DL tasks (Figure 5). In the DL task (Figure 5A), ASSR modulation in the left and right AC was not significantly correlated with the left or right ears (left ear: left AC: *R*^2^ = 0.058, *p* = 0.750, right AC: *R*^2^ = 0.007, *p* = 0.335; right ear: left AC: *R*^2^ = 0.007, *p* = 0.740, right AC: *R*^2^ = 0.045, *p* = 0.400). In the diotic listening task (Figure 5B), ASSR modulation in the right ear in the left AC tended to correlate with accuracy during the diotic listening task (*R*^2^ = 0.209, *p* = 0.055). In the left ear no significant correlation between accuracy and ASSR modulation was observed (left AC: *R*^2^ = 0.001, *p* = 0.894, right AC: *R*^2^ = 0.077, *p* = 0.264).

**Figure 6:**
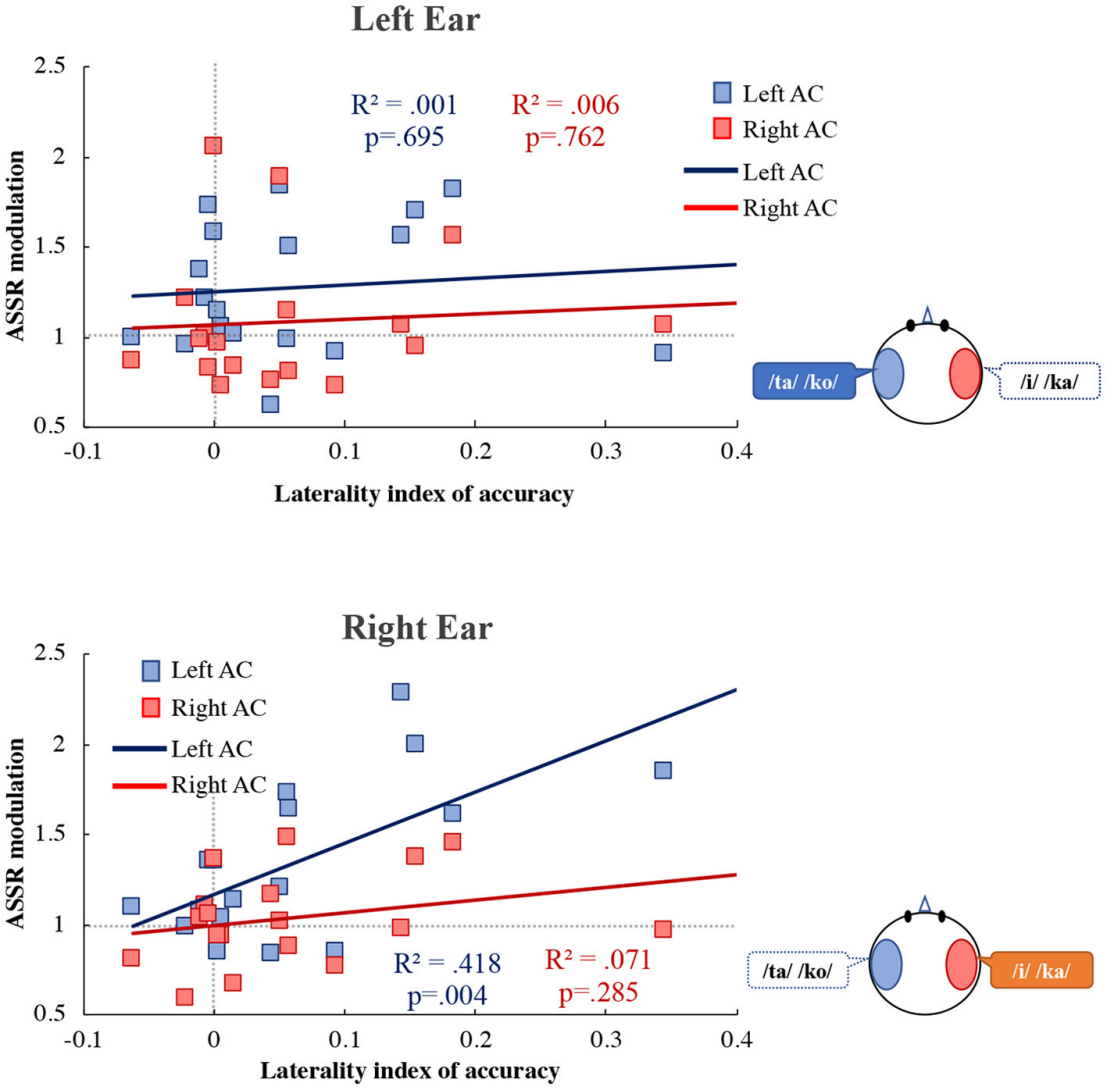
Correlation between the laterality index of accuracy and the ASSR modulation in the left (blue) and right (red) auditory cortex (Left AC or Right AC) to the left (upper figure) and right (lower figure) ears during the dichotic listening task. X-axis shows the laterality index of accuracy. Y-axis shows the ASSR modulation. The more to the right, more REA in the accuracy is indicated, and to the left, the LEA is indicated. *R*^2^ indicates determination coefficient. ASSR, auditory steady-state response; AC, auditory cortex.

Figure 6 shows the correlation between the LI of accuracy and ASSR modulation. In the right ear, ASSR modulation was significantly correlated with the LI of accuracy in the left AC (*R*^2^ = 0.418, *p* = 0.004). However, there was no significant correlation between the ASSR modulation and the LI of accuracy in the right AC (*R*^2^ = 0.071, *p* = 0.285). ANCOVA was performed to determine whether the left and right AC differed. The analysis revealed a significant main effect of hemisphere [*F*_(1, 32)_ = 9.38, *p* = 0.004] and accuracy [*F*_(1, 32)_ = 11.74, *p* = 0.002]. There was a significant interaction effect between the hemispheres and accuracy [*F*_(1, 32)_ = 4.35, *p* = 0.045], indicating that the ASSR modulation differed significantly between the left and right AC. We also performed a statistical test for equality of the regression coefficients (Paternoster et al., 1998). As a result, there was a significant difference in the correlation coefficient between the left AC and the right AC of the right ear (right ear: *z* = 3.24, *p* = 0.005, left ear: *z* = 0.18, *p* = 0.858). In the left ear, there was no significant correlation between the ASSR modulation and the LI of accuracy in the left AC (*R*^2^ = 0.001, *p* = 0.695) and right AC (*R*^2^ = 0.006, *p* = 0.762).

## Discussion

Listeners are more likely to report speech presented to the right ear than speech presented to the left ear (REA) (Kimura, 1961a,b, 1967; Jäncke et al., 2001; Alho et al., 2012; Payne et al., 2017). However, listeners do not pay 100% attention to the right ear. Therefore, several questions remain unanswered regarding REA in the existent literature (Kimura, 1961a,b; Jäncke et al., 2001; Alho et al., 2012; Payne et al., 2017). Specifically, which ear is used for attention and what is the ratio of attention distribution between the right and left ears? We assessed the relationship between individual accuracy and ASSR modulation. In previous studies on the REA, directed DL tasks have been used, in which participants are instructed to pay attention to the left or right ear (Jäncke et al., 2001; Alho et al., 2012; Payne et al., 2017). In this study, we used a free-reported DL task (non-instruction) and two-syllable Japanese meaningful words to simulate real-life perceptions as much as possible. Therefore, our DL task may be more difficult than the directed DL task, and the ceiling effect of accuracy can be eliminated. Thus, we were able to determine the correlation between accuracy and ASSR modulation. With regard to our behavioral results, the mean accuracy was 64.15% (SD = 15.72%) and 73.61% (SD = 14.93%) in the left and the right ears, respectively, during the DL task (Figure 2), demonstrating the REA. In addition, it was confirmed that the accuracy was not affected by the modulation frequency. As a result, it was shown that the frequency tagging method used in this study can be applied to the DL task. In the following, we interpret our neurophysiological findings according to the structure and attention models of the REA in DL (Hiscock and Kinsbourne, 2011).

### The Structure Model: Left Hemispheric Dominance

The majority of people have dominantly left hemispheric language representation and show right-ear dominance when reporting speech presented in the DL task. Therefore, among most people, the neural pathway from the right ear to the left hemisphere is presumed to be superior to the pathway from the left ear to the left hemisphere (contralateral projection dominance) (Hiscock and Kinsbourne, 2011). The structural model of the REA combined these two elements. The relationship between REA and left hemispheric language representation has also been demonstrated by invasive and non-invasive methods (Geffen and Caudrey, 1981; Zatorre, 1989; Hugdahl et al., 1997).

In this study, the ASSR amplitude in the left AC was significantly larger than that in the right AC in the active condition during the DL and diotic listening tasks; in particular, the response to right ear stimuli in the DL task displayed left hemispheric dominance (Figure 3A). Additionally, the ASSR modulation in the left AC was significantly larger than that in the right AC in the DL task (Figure 4A). Alternatively, no difference was observed between the ASSR modulation in the left AC to the left and right ears during the DL and diotic listening tasks (Figure 4). Therefore, contralateral projection dominance was not observed in the present study. The structural model does not provide an explanation for the ability of cued or directed DL in the left ear to overcome the REA or why attention directed to the right ear can amplify the REA (Payne et al., 2017). Therefore, we consider whether the rightward attentional bias in the DL task can be explained by the ASSR modulation used in this study.

### The Attention Model: Rightward Attention Bias

Our study was designed to investigate top-down functions, such as ASSR modulation. Previous studies on the effects of attention have been controversial. Some reports have shown that increasing attention to a stimulus enhances the 40-Hz ASSRs (Ross et al., 2004; Skosnik et al., 2007; Saupe et al., 2009; Gander et al., 2010; Herdman, 2011), while others have reported no effects on attention (Linden et al., 1987; de Jong et al., 2010; Mahajan et al., 2014). A possible reason for this discrepancy is that these studies could not clearly discriminate between bottom-up and top-down functions. The 40-Hz ASSR amplitude is modulated by varying stimulation properties (bottom-up) and paying attention to the sounds (top-down) during the DL task. To detect the top-down effect, we measured the ASSR in the passive condition and examined non-specific binding circuit activity in the 40-Hz gamma oscillation network (Ross and Fujioka, 2016) as ASSR modulation by removing bottom-up effects from the ASSR amplitude. To determine whether the 40-Hz ASSR is reflective of the gamma oscillatory mechanism, it was useful to show that evoked and induced gamma oscillations in a perception-related study clearly demonstrated the generation of the gamma oscillatory mechanism (Bidet-Caulet et al., 2007; Cardin et al., 2009). Gamma oscillations have been suggested to play an important role at the sensory level and beyond for higher-order perception (Ross and Fujioka, 2016). The 40-Hz ASSR is closely involved in the gamma oscillation in thalamocortical networks, and the direct link between gamma oscillations and the 40-Hz ASSR has been reported in previous studies (Basar, 2013; O'Donnell et al., 2013).

During the DL task, ASSR modulation in the left AC was larger than that in the right AC (Figure 4A). Consistent with our results, Ross et al. (2004) reported that 40-Hz ASSR was enhanced in the contralateral left hemisphere following right ear stimulation. Müller et al. (2009) pointed out that 20-Hz ASSR modulation in the left hemisphere has complex effects on attention, and that contralateral activation is enhanced while ipsilateral activation is reduced. Information from the ipsilateral ear during DL is relatively suppressed compared to information from the contralateral ear (Brancucci et al., 2004). However, our results did not indicate suppression of unattended information processing. In previous studies, directed DL tasks were used, in which participants were instructed to pay attention to the left or right ear (Jäncke et al., 2001; Alho et al., 2012; Payne et al., 2017). Therefore, the suppression of unattended information processing may be relatively easily observed as brain activity. Since participants in our study were required to pay attention to both ears during our tasks, more complicated changes in brain activity were observed compared to previous studies. In this study, the ASSR amplitude is mainly related to stimulus characteristics (bottom-up), was not significantly different between the DL and the diotic listening tasks. On the other hand, the ASSR amplitude in the active condition was significantly larger than in the passive condition (Figure 3). Therefore, ASSR modulation may be related to tasks that require active attention. Our results of ASSR modulations during the DL task were not correlated with the accuracy in each ear (Figure 5A). However, the ASSR modulation in the left AC was significantly correlated with the LI of accuracy in the right ear (Figure 6). Our observation of the 40-Hz ASSR modulation during the DL task suggests that it may be related to auditory allocation of attention.

These results suggest that ASSR modulation is not related to the accuracy in each ear, but rather to the ratio of attention to each ear. Payne et al. (2017) recently explored the relationship between LI in alpha power and behavioral results and did not detect any significant correlations. To our knowledge, no previous study has separated ipsilateral and contralateral activity and quantitatively assessed the relationship between the ratio of paying attention and the 40-Hz ASSR modulation.

During the diotic listening task, we did not observe any difference in ASSR modulation between the left and right AC (Figure 4B). However, ASSR modulation in the right ear of the left AC tended to correlate with accuracy (Figure 5B). When the same speech stimulus is presented to the left and right ears, we do not know which ear the participant is paying attention to when responding. However, the correlations in the results of Figure 5B suggest that the participants may be paying more attention to the speech input from the right ear than the left ear. The 40-Hz ASSR modulation can aid in understanding the connection between the allocation of attention and the REA. Thus, the 40-Hz ASSR modulation may determine differences in attention to the left and right ears.

In conclusion, our results revealed a correlation between the attention-related increase in the amplitude modulation of ASSRs and the LI of behavioral accuracy. However, given that the participants of this study were healthy adult undergraduate and graduate students, the accuracy in both ears in the DL was high, and few participants displayed a clear REA. Therefore, in Figure 6, there are few plots of data showing the REAs with a higher LI of accuracy. By increasing the number of participants in the study and including a wide range of age groups, we expect to obtain a more pronounced REA and a clear, high index of correlation between the ASSR modulation and the LI of accuracy. The DL test is one of the most frequently included tests in the battery for the diagnosis of auditory processing disorder (APD) in children. If the relationship between attentional distribution and cerebral neural activity in REA can be quantitatively assessed, it may help in the diagnosis of the APD and other auditory disorders associated with central functions.

## Data Availability Statement

The raw data supporting the conclusions of this article will be made available by the authors, without undue reservation.

## Ethics Statement

The studies involving human participants were reviewed and approved by Research Ethics Committee of Tokyo Denki University. The patients/participants provided their written informed consent to participate in this study.

## Author Contributions

KT and SK conceived and designed the study. BR designed the analysis methods for this study. TH and CO recorded and provided the experimental sources. KT coordinated the acquisition of data, analyzed the data, and drafted the manuscript. All authors contributed to the article and approved the submitted version.

## Conflict of Interest

The authors declare that the research was conducted in the absence of any commercial or financial relationships that could be construed as a potential conflict of interest.

## Figures and Tables

**Appendix 1:**
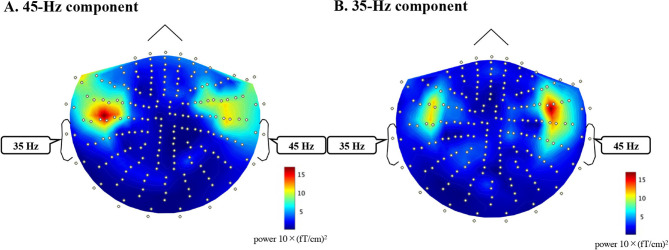
Topography of 45-Hz **(A)** and 35-Hz **(B)** components of FFT analysis (0–500 ms) of the grand averaged waveform of all subjects during a dichotic listening task (left ear mf = 35 Hz, right ear mf = 45 Hz).
